# 3-(1*H*-Indol-3-yl)-2-(2-nitro­benzene­sulfonamido)­propanoic acid including an unknown solvate

**DOI:** 10.1107/S1600536812023446

**Published:** 2012-06-13

**Authors:** Islam Ullah Khan, Hafiz Mubashar-ur-Rehman, Salman Aziz, William T. A. Harrison

**Affiliations:** a Materials Chemistry laboratory, Department of Chemistry, GC University, Lahore 54000, Pakistan; b Department of Chemistry, University of Aberdeen, Aberdeen AB24 3UE, Scotland

## Abstract

In the title compound, C_17_H_15_N_3_O_6_S, which crystallized with highly disordered methanol and/or water solvent mol­ecules, the dihedral angle between the the indole and benzene ring systems is 5.3 (2)°, which allows for the formation of intra­molecular π–π stacking inter­actions [centroid–centroid separations = 3.641 (3) and 3.694 (3) Å] and an approximate overall U-shape for the mol­ecule. In the crystal, dimers linked by pairs of N_s_—H⋯O_c_ (s = sulfonamide and c = carboxyl­ate) hydrogen bonds generate *R*_2_^2^(10) loops, whereas N_i_—H⋯π (i = indole) inter­actions lead to chains propagating in [100] or [010]. Together, these lead to a three-dimensional network in which the solvent voids are present as inter­secting (two-dimensional) systems of [100] and [010] channels. The title compound was found to contain a heavily disordered solvent mol­ecule, which could be methanol or water or a mixture of the two. Due to its uncertain nature and the unresolvable disorder, the data were processed with the SQUEEZE option in *PLATON* [Spek (2009 ▶). *Acta Cryst.* D**65**, 148–155], which revealed 877.8 Å^3^ of solvent-accessible volume per unit cell and 126 electron-units of scattering density or 109.7 Å^3^ (16 electron units) per organic mol­ecule.. This was not included in the calculations of overall formula weight, density and absorption coefficient.

## Related literature

For related structures and background references to the biological activity of sulfonamides, see: Khan *et al.* (2011*a* ▶,*b* ▶). For further synthetic details, see: Deng & Mani (2006 ▶).
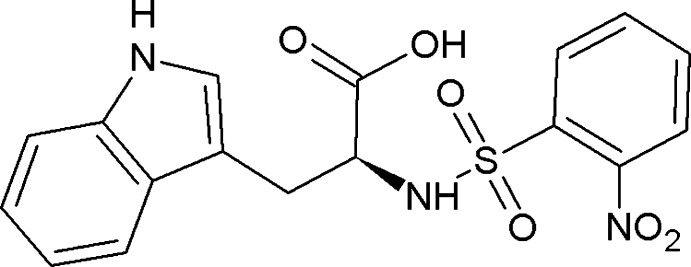


## Experimental

### 

#### Crystal data

C_17_H_15_N_3_O_6_S
*M* *_r_* = 389.38
Tetragonal, 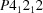

*a* = 9.6818 (5) Å
*c* = 44.017 (3) Å
*V* = 4126.0 (4) Å^3^
*Z* = 8
Mo *K*α radiation
μ = 0.19 mm^−1^
*T* = 296 K
0.30 × 0.25 × 0.10 mm

#### Data collection

Bruker Kappa APEXII CCD diffractometer
4042 measured reflections
4042 independent reflections
3492 reflections with *I* > 2σ(*I*)

#### Refinement

*R*[*F* ^2^ > 2σ(*F* ^2^)] = 0.067
*wR*(*F* ^2^) = 0.168
*S* = 1.07
4042 reflections
245 parameters
H-atom parameters constrained
Δρ_max_ = 0.21 e Å^−3^
Δρ_min_ = −0.24 e Å^−3^
Absolute structure: Flack (1983 ▶), 1581 Friedel pairs
Flack parameter: 0.03 (15)

### 

Data collection: *APEX2* (Bruker, 2007 ▶); cell refinement: *SAINT* (Bruker, 2007 ▶); data reduction: *SAINT*; program(s) used to solve structure: *SHELXS97* (Sheldrick, 2008 ▶); program(s) used to refine structure: *SHELXL97* (Sheldrick, 2008 ▶); molecular graphics: *ORTEP-3* (Farrugia, 1997 ▶); software used to prepare material for publication: *SHELXL97* and *PLATON* (Spek, 2009 ▶).

## Supplementary Material

Crystal structure: contains datablock(s) I, global. DOI: 10.1107/S1600536812023446/sj5218sup1.cif

Structure factors: contains datablock(s) I. DOI: 10.1107/S1600536812023446/sj5218Isup2.hkl

Supplementary material file. DOI: 10.1107/S1600536812023446/sj5218Isup3.cml

Additional supplementary materials:  crystallographic information; 3D view; checkCIF report

## Figures and Tables

**Table 1:** Hydrogen-bond geometry (Å, °)

*D*—H⋯*A*	*D*—H	H⋯*A*	*D*⋯*A*	*D*—H⋯*A*
N1—H1⋯*Cg*1^i^	0.86	2.77	3.565 (4)	155
N2—H2⋯O1^ii^	0.86	2.10	2.918 (4)	158

Symmetry codes: (i) 
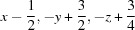
; (ii) 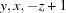.

**Fig. 1.**
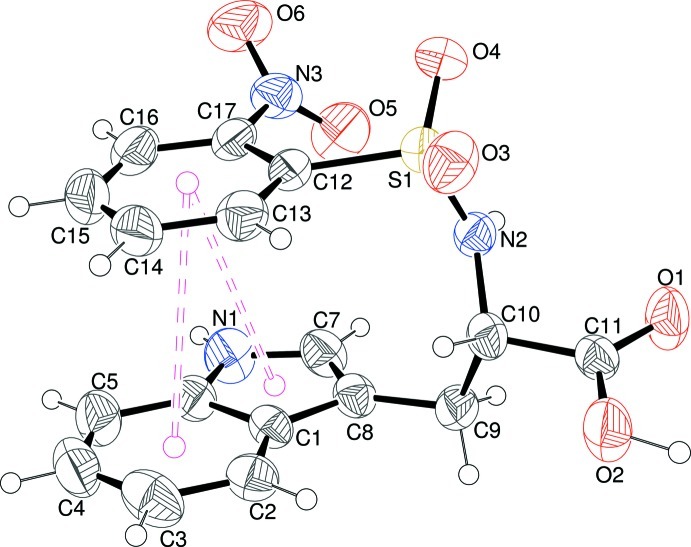
The molecular structure of (I) with displacement ellipsoids drawn at the 40% probability level and the intramolecular π-π stacking interactions shown as double-dashed lines betweent the ring centroids.

**Fig. 2.**
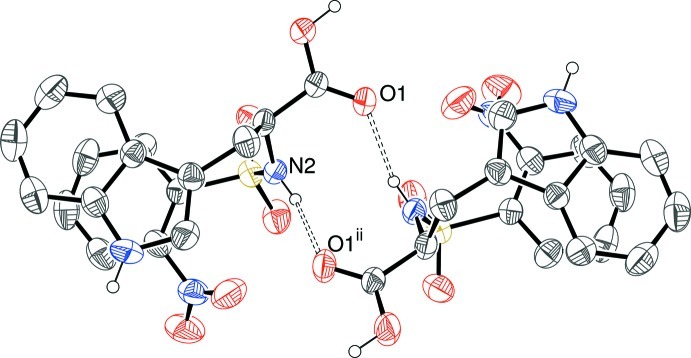
Detail of the structure of (I) showing the formation of dimers linked by pairs of N—H···O hydrogen bonds, thus generating R22(10) loops. All C-bonded H atoms omitted for clarity. Symmetry code: (ii) y, x, 1 - z.

**Fig. 3.**
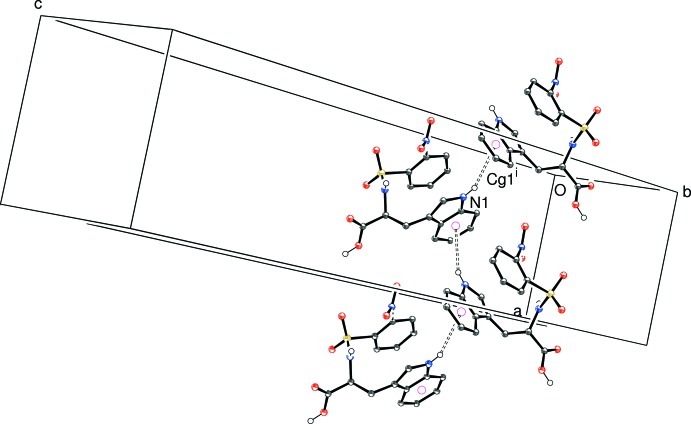
Detail of the structure of (I) showing the formation of [100] chains linked by N—H···π interactions. Cg1 (pink circle) is the centroid of the C1–C6 ring. Symmetry code: (i) x - 1/2, 3/2 - y, 3/4 - z.

**Fig. 4.**
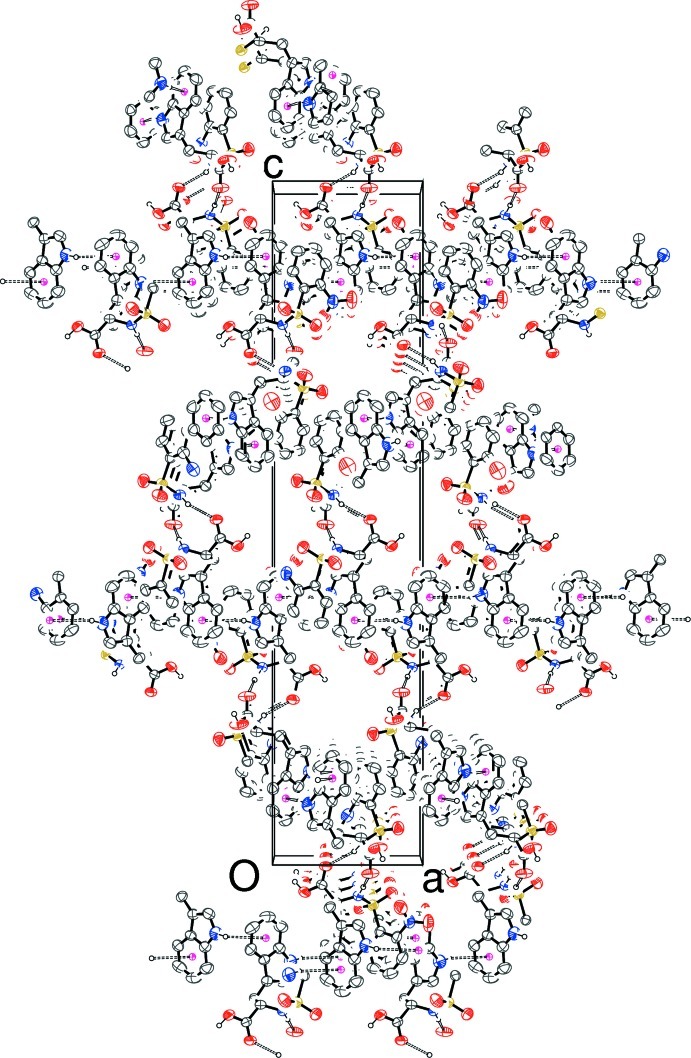
The unit-cell packing for (I) viewed approximately down [010] showing the solvent voids.

## supplementary crystallographic information

### Comment

As part of our ongoing studies of chiral sulfonamides with possible biological activity (Khan *et al.*, 2011*a*,*b*), we now report the structure of the title compound, (I). Compound (I) was found to contain a heavily disordered solvent molecule, which could be methanol or water or a mixture of the two. Due to its uncertain nature and the unresolvable disorder, the data were processed with the SQUEEZE option in *PLATON* (Spek, 2009), to remove the solvent contribution to the scattering.

The molecular structure of (I) (Fig. 1) approximates to a U-shape, with the indole ring system (C1—C8/N1; r.m.s. deviation = 0.007 Å) and benzene ring (C12–C17) lying approximately parallel to each other [dihedral angle = 5.3 (2)°]. This allows intramolecular aromatic π-π stacking to occur: the separations of the centroid of the C12–C17 benzene ring with those of the C1–C6 and C1/C6/C7/C8/N1 rings are 3.641 (3) Å and 3.694 (3) Å, respectively. The N3/O5/O6 nitro group is twisted out of the plane of its atttached ring by 48.9 (4)°. The configuration of the stereogenic carbon atom (C10) in (I) is S, which is consistent with that of the equivalent atom in the starting material.

In the crystal, the molecules are linked into dimers *via* pairs of N_s_–H···O_c_ (s = sulfonamide, c = carboxylate) hydrogen bonds (Fig. 2, Table 1), which result in *R*_2_^2^(10) loops. A crystallographic twofold axis directed along [110] generates the second molecule from the asymmetric molecule. In addition, weak N_i_—H···π (i = indole) interactions occur: these lead to [100] chains for the asymmetric molecule and [010] chains for symmetry-generated molecules in other locations in the unit-cell (Fig. 3). The carboxylic acid O—H group is directed towards the solvent void and probably forms a hydrogen bond to the solvent.

Together, the N–H···O and N–H···π bonds generate a three-dimensional network of molecules within the distinctive "tall" tetragonal unit-cell (Fig. 3). The solvent voids are apparent as square grids of intersecting [100] and [010] pseudo channels lying at *z* = 0, *z* = 1/4 and symmetry equivalent locations.

The molcular conformation and crystal structure (Khan *et al.*, 2011*a*) of the closely related compound 3-(1*H*-indol-3-yl)-2-(toluene-4-sulfonylamino)-propionic acid monohydrate, (II), are completely different to (I). In (II), where a *para*-toluene substituent has replaced the 2-nitrobebzene substituent in (I), the organic molecule adopts an extended *Z*-shaped conformation and no intramolecular π-π stacking can occur. In the crystal of (II), in which the solvent water molecule was located, N_s_–H···O_s_ hydrogen bonds and O_c_–H···O_w_ (s = sulfonamide, c = carboxylic acid, w = water) hydrogen bonds generate chains and the crystal symmetry is monoclinic. Another feature of (II) not seen in (I) is the presence of a short intermolecular C—H···O interaction arising from the α (chiral) C atom (Khan *et al.*, 2011*b*). However, it is interesting to note that (I) and (II) both feature an unusual N_i_–H···π (i = indole) interaction.

### Experimental

The title compound was prepared following the literature method (Deng & Mani, 2006) and recrystalized from methanol by slow evaporation to yield yellow blocks of (I).

### Refinement

Due to the disordered solvent molecule and its uncertain identity, the data were processed with SQUEEZE in *PLATON* (Spek, 2009). This revealed 877.8 Å^3^ of solvent accessible volume per unit cell and 126 electron-units of scattering density or 109.7 Å^3^ (16 electron units) per organic molecule. This was not included in the calculations of overall formula weight, density and absorption coefficient. The original data set consisted of 31099 measured reflections (-11 ≤ h ≤ 11, -11 ≤ k ≤ 11, -54 ≤ l ≤ 54), for which R_int_ was 0.068.

The C- and N-bound H-atoms were geometrically placed (C—H = 0.93–0.98 Å, N—H = 0.86 Å) and refined as riding with *U*_iso_(H) = 1.2*U*_eq_(carrier). The O-bound H was located in a difference map and refined as riding in its as-found relative position with *U*_iso_(H) = 1.5*U*_eq_(O).

### Crystal data

**Table:**

C_17_H_15_N_3_O_6_S	*D*_x_ = 1.254 Mg m^−^^3^
*M**_r_* = 389.38	Mo *K*α radiation, λ = 0.71073 Å
Tetragonal, *P*4_1_2_1_2	Cell parameters from 9980 reflections
Hall symbol: P 4abw 2nw	θ = 2.8–26.9°
*a* = 9.6818 (5) Å	µ = 0.19 mm^−^^1^
*c* = 44.017 (3) Å	*T* = 296 K
*V* = 4126.0 (4) Å^3^	Block, yellow
*Z* = 8	0.30 × 0.25 × 0.10 mm
*F*(000) = 1616	

### Data collection

**Table:**

Bruker Kappa APEXII CCD diffractometer	3492 reflections with *I* > 2σ(*I*)
Radiation source: fine-focus sealed tube	*R*_int_ = 0.000
Graphite monochromator	θ_max_ = 26.0°, θ_min_ = 2.5°
ω scans	*h* = −7→8
4042 measured reflections	*k* = 0→11
4042 independent reflections	*l* = 0→54

### Refinement

**Table:**

Refinement on *F*^2^	Hydrogen site location: inferred from neighbouring sites
Least-squares matrix: full	H-atom parameters constrained
*R*[*F*^2^ > 2σ(*F*^2^)] = 0.067	*w* = 1/[σ^2^(*F*_o_^2^) + (0.0679*P*)^2^ + 3.5726*P*] where *P* = (*F*_o_^2^ + 2*F*_c_^2^)/3
*wR*(*F*^2^) = 0.168	(Δ/σ)_max_ < 0.001
*S* = 1.07	Δρ_max_ = 0.21 e Å^−^^3^
4042 reflections	Δρ_min_ = −0.24 e Å^−^^3^
245 parameters	Extinction correction: *SHELXL*, Fc^*^=kFc[1+0.001xFc^2^λ^3^/sin(2θ)]^-1/4^
0 restraints	Extinction coefficient: 0.0083 (12)
Primary atom site location: structure-invariant direct methods	Absolute structure: Flack (1983), 1581 Friedel pairs
Secondary atom site location: difference Fourier map	Flack parameter: 0.03 (15)

### Special details

**Table:**

Geometry. All e.s.d.'s (except the e.s.d. in the dihedral angle between two l.s. planes) are estimated using the full covariance matrix. The cell e.s.d.'s are taken into account individually in the estimation of e.s.d.'s in distances, angles and torsion angles; correlations between e.s.d.'s in cell parameters are only used when they are defined by crystal symmetry. An approximate (isotropic) treatment of cell e.s.d.'s is used for estimating e.s.d.'s involving l.s. planes.
Refinement. Refinement of *F*^2^ against ALL reflections. The weighted *R*-factor *wR* and goodness of fit *S* are based on *F*^2^, conventional *R*-factors *R* are based on *F*, with *F* set to zero for negative *F*^2^. The threshold expression of *F*^2^ > σ(*F*^2^) is used only for calculating *R*-factors(gt) *etc*. and is not relevant to the choice of reflections for refinement. *R*-factors based on *F*^2^ are statistically about twice as large as those based on *F*, and *R*- factors based on ALL data will be even larger.

### Fractional atomic coordinates and isotropic or equivalent isotropic displacement parameters (Å^2^)

**Table:**

	*x*	*y*	*z*	*U*_iso_*/*U*_eq_	
C1	0.5558 (4)	0.6025 (4)	0.39025 (8)	0.0488 (8)	
C2	0.6443 (5)	0.5208 (5)	0.37363 (10)	0.0583 (10)	
H2A	0.7108	0.4675	0.3834	0.070*	
C3	0.6335 (6)	0.5186 (6)	0.34215 (11)	0.0780 (15)	
H3A	0.6925	0.4627	0.3309	0.094*	
C4	0.5350 (7)	0.5994 (6)	0.32731 (10)	0.0792 (15)	
H4	0.5306	0.5977	0.3062	0.095*	
C5	0.4460 (6)	0.6799 (6)	0.34293 (11)	0.0763 (14)	
H5	0.3799	0.7328	0.3329	0.092*	
C6	0.4564 (5)	0.6813 (4)	0.37487 (9)	0.0558 (10)	
C7	0.4312 (5)	0.7179 (5)	0.42414 (10)	0.0641 (11)	
H7	0.3953	0.7511	0.4423	0.077*	
C8	0.5374 (4)	0.6301 (4)	0.42204 (8)	0.0505 (9)	
C9	0.6174 (4)	0.5663 (4)	0.44725 (9)	0.0554 (10)	
H9A	0.6013	0.6180	0.4658	0.067*	
H9B	0.7152	0.5717	0.4426	0.067*	
C10	0.5775 (4)	0.4149 (4)	0.45240 (8)	0.0500 (9)	
H10	0.5916	0.3639	0.4334	0.060*	
C11	0.6702 (4)	0.3540 (4)	0.47649 (8)	0.0481 (9)	
C12	0.2877 (4)	0.3494 (4)	0.40872 (8)	0.0467 (8)	
C13	0.3606 (5)	0.2926 (5)	0.38483 (9)	0.0612 (11)	
H13	0.4322	0.2310	0.3887	0.073*	
C14	0.3275 (6)	0.3271 (6)	0.35519 (9)	0.0734 (14)	
H14	0.3782	0.2905	0.3391	0.088*	
C15	0.2204 (7)	0.4150 (6)	0.34959 (10)	0.0777 (14)	
H15	0.1990	0.4378	0.3296	0.093*	
C16	0.1439 (5)	0.4701 (6)	0.37262 (11)	0.0718 (13)	
H16	0.0696	0.5281	0.3686	0.086*	
C17	0.1800 (4)	0.4373 (4)	0.40226 (9)	0.0552 (10)	
S1	0.33465 (11)	0.29054 (11)	0.44601 (2)	0.0528 (3)	
N1	0.3822 (4)	0.7526 (4)	0.39580 (9)	0.0713 (11)	
H1	0.3162	0.8094	0.3920	0.086*	
N2	0.4319 (3)	0.4032 (3)	0.46119 (6)	0.0484 (7)	
H2	0.3988	0.4579	0.4747	0.058*	
N3	0.0981 (4)	0.5018 (4)	0.42620 (10)	0.0664 (10)	
O1	0.6449 (3)	0.3541 (4)	0.50311 (6)	0.0781 (10)	
O2	0.7836 (3)	0.3064 (4)	0.46516 (6)	0.0853 (12)	
H3	0.8481	0.2766	0.4797	0.102*	
O3	0.4148 (3)	0.1700 (3)	0.44107 (7)	0.0718 (9)	
O4	0.2103 (3)	0.2828 (4)	0.46327 (7)	0.0755 (10)	
O5	0.1554 (4)	0.5580 (5)	0.44684 (9)	0.0921 (12)	
O6	−0.0272 (4)	0.4964 (5)	0.42333 (11)	0.0987 (13)	

### Atomic displacement parameters (Å^2^)

**Table:**

	*U*^11^	*U*^22^	*U*^33^	*U*^12^	*U*^13^	*U*^23^
C1	0.055 (2)	0.044 (2)	0.0473 (18)	−0.0035 (17)	0.0050 (17)	0.0033 (16)
C2	0.059 (3)	0.053 (2)	0.062 (2)	−0.005 (2)	0.006 (2)	−0.0003 (19)
C3	0.096 (4)	0.071 (3)	0.068 (3)	−0.006 (3)	0.028 (3)	−0.012 (2)
C4	0.112 (5)	0.075 (3)	0.050 (2)	−0.024 (3)	−0.001 (3)	0.007 (2)
C5	0.087 (4)	0.078 (3)	0.064 (3)	−0.005 (3)	−0.012 (3)	0.021 (3)
C6	0.062 (3)	0.049 (2)	0.057 (2)	−0.004 (2)	−0.0034 (18)	0.0095 (18)
C7	0.068 (3)	0.068 (3)	0.056 (2)	0.005 (2)	0.011 (2)	0.004 (2)
C8	0.057 (2)	0.044 (2)	0.0509 (19)	−0.0017 (17)	0.0004 (17)	0.0059 (16)
C9	0.057 (2)	0.056 (2)	0.053 (2)	−0.0113 (18)	−0.0096 (18)	0.0039 (18)
C10	0.054 (2)	0.062 (2)	0.0342 (15)	0.0002 (19)	−0.0001 (15)	−0.0023 (15)
C11	0.049 (2)	0.055 (2)	0.0394 (16)	0.0018 (17)	0.0053 (15)	0.0022 (15)
C12	0.046 (2)	0.049 (2)	0.0459 (17)	−0.0078 (17)	−0.0033 (16)	−0.0022 (15)
C13	0.060 (3)	0.066 (3)	0.057 (2)	−0.007 (2)	0.0011 (19)	−0.013 (2)
C14	0.085 (3)	0.086 (3)	0.050 (2)	−0.031 (3)	0.006 (2)	−0.010 (2)
C15	0.099 (4)	0.082 (4)	0.051 (2)	−0.028 (3)	−0.015 (3)	0.004 (2)
C16	0.058 (3)	0.081 (3)	0.077 (3)	−0.007 (2)	−0.019 (2)	0.013 (2)
C17	0.044 (2)	0.060 (2)	0.062 (2)	−0.0064 (19)	−0.0104 (18)	0.0037 (19)
S1	0.0515 (6)	0.0560 (6)	0.0508 (5)	−0.0032 (5)	−0.0041 (4)	0.0055 (4)
N1	0.070 (3)	0.064 (2)	0.080 (2)	0.0302 (19)	0.002 (2)	0.0077 (19)
N2	0.0449 (17)	0.060 (2)	0.0401 (14)	0.0036 (15)	0.0014 (13)	−0.0026 (14)
N3	0.050 (2)	0.069 (3)	0.081 (2)	0.0070 (18)	0.0047 (19)	0.014 (2)
O1	0.068 (2)	0.128 (3)	0.0381 (13)	0.030 (2)	0.0032 (13)	0.0059 (16)
O2	0.0609 (19)	0.147 (4)	0.0478 (14)	0.039 (2)	0.0108 (14)	0.0140 (19)
O3	0.076 (2)	0.0584 (18)	0.081 (2)	0.0064 (17)	−0.0180 (17)	−0.0001 (16)
O4	0.0590 (18)	0.102 (3)	0.0655 (17)	−0.0225 (18)	0.0054 (15)	0.0188 (18)
O5	0.075 (2)	0.118 (3)	0.082 (2)	0.013 (2)	0.010 (2)	−0.031 (2)
O6	0.046 (2)	0.115 (3)	0.135 (3)	0.010 (2)	0.000 (2)	0.015 (3)

### Geometric parameters (Å, º)

**Table:**

C1—C2	1.377 (6)	C11—O1	1.197 (4)
C1—C6	1.402 (6)	C11—O2	1.291 (5)
C1—C8	1.436 (5)	C12—C17	1.375 (5)
C2—C3	1.390 (7)	C12—C13	1.381 (6)
C2—H2A	0.9300	C12—S1	1.796 (4)
C3—C4	1.396 (8)	C13—C14	1.384 (6)
C3—H3A	0.9300	C13—H13	0.9300
C4—C5	1.350 (8)	C14—C15	1.363 (8)
C4—H4	0.9300	C14—H14	0.9300
C5—C6	1.410 (6)	C15—C16	1.364 (8)
C5—H5	0.9300	C15—H15	0.9300
C6—N1	1.356 (6)	C16—C17	1.387 (6)
C7—C8	1.337 (6)	C16—H16	0.9300
C7—N1	1.376 (6)	C17—N3	1.459 (6)
C7—H7	0.9300	S1—O3	1.418 (3)
C8—C9	1.488 (5)	S1—O4	1.426 (3)
C9—C10	1.533 (6)	S1—N2	1.588 (3)
C9—H9A	0.9700	N1—H1	0.8600
C9—H9B	0.9700	N2—H2	0.8600
C10—N2	1.466 (5)	N3—O5	1.195 (5)
C10—C11	1.509 (5)	N3—O6	1.221 (5)
C10—H10	0.9800	O2—H3	0.9400
			
C2—C1—C6	118.9 (4)	O1—C11—C10	124.5 (4)
C2—C1—C8	134.6 (4)	O2—C11—C10	111.9 (3)
C6—C1—C8	106.5 (4)	C17—C12—C13	118.5 (4)
C1—C2—C3	119.5 (5)	C17—C12—S1	125.2 (3)
C1—C2—H2A	120.3	C13—C12—S1	116.1 (3)
C3—C2—H2A	120.3	C12—C13—C14	120.2 (5)
C2—C3—C4	120.6 (5)	C12—C13—H13	119.9
C2—C3—H3A	119.7	C14—C13—H13	119.9
C4—C3—H3A	119.7	C15—C14—C13	119.8 (5)
C5—C4—C3	121.5 (4)	C15—C14—H14	120.1
C5—C4—H4	119.3	C13—C14—H14	120.1
C3—C4—H4	119.3	C14—C15—C16	121.5 (4)
C4—C5—C6	117.9 (5)	C14—C15—H15	119.2
C4—C5—H5	121.1	C16—C15—H15	119.2
C6—C5—H5	121.1	C15—C16—C17	118.2 (5)
N1—C6—C1	108.2 (3)	C15—C16—H16	120.9
N1—C6—C5	130.1 (4)	C17—C16—H16	120.9
C1—C6—C5	121.7 (4)	C12—C17—C16	121.8 (4)
C8—C7—N1	110.9 (4)	C12—C17—N3	121.8 (4)
C8—C7—H7	124.5	C16—C17—N3	116.4 (4)
N1—C7—H7	124.5	O3—S1—O4	120.0 (2)
C7—C8—C1	106.3 (4)	O3—S1—N2	107.77 (18)
C7—C8—C9	127.8 (4)	O4—S1—N2	108.22 (19)
C1—C8—C9	125.8 (4)	O3—S1—C12	105.04 (19)
C8—C9—C10	112.1 (3)	O4—S1—C12	106.84 (19)
C8—C9—H9A	109.2	N2—S1—C12	108.47 (17)
C10—C9—H9A	109.2	C6—N1—C7	108.0 (4)
C8—C9—H9B	109.2	C6—N1—H1	126.0
C10—C9—H9B	109.2	C7—N1—H1	126.0
H9A—C9—H9B	107.9	C10—N2—S1	120.8 (3)
N2—C10—C11	110.9 (3)	C10—N2—H2	119.6
N2—C10—C9	110.8 (3)	S1—N2—H2	119.6
C11—C10—C9	109.1 (3)	O5—N3—O6	124.0 (5)
N2—C10—H10	108.7	O5—N3—C17	119.4 (4)
C11—C10—H10	108.7	O6—N3—C17	116.6 (5)
C9—C10—H10	108.7	C11—O2—H3	114.3
O1—C11—O2	123.5 (4)		
			
C6—C1—C2—C3	0.2 (6)	C12—C13—C14—C15	−1.5 (7)
C8—C1—C2—C3	−179.9 (5)	C13—C14—C15—C16	−0.2 (8)
C1—C2—C3—C4	0.7 (7)	C14—C15—C16—C17	1.6 (8)
C2—C3—C4—C5	−1.2 (8)	C13—C12—C17—C16	−0.2 (6)
C3—C4—C5—C6	0.7 (8)	S1—C12—C17—C16	−175.2 (4)
C2—C1—C6—N1	−179.9 (4)	C13—C12—C17—N3	−179.9 (4)
C8—C1—C6—N1	0.1 (5)	S1—C12—C17—N3	5.2 (6)
C2—C1—C6—C5	−0.7 (6)	C15—C16—C17—C12	−1.4 (7)
C8—C1—C6—C5	179.4 (4)	C15—C16—C17—N3	178.2 (4)
C4—C5—C6—N1	179.3 (5)	C17—C12—S1—O3	160.3 (4)
C4—C5—C6—C1	0.2 (7)	C13—C12—S1—O3	−14.8 (4)
N1—C7—C8—C1	−1.9 (5)	C17—C12—S1—O4	31.7 (4)
N1—C7—C8—C9	−178.9 (4)	C13—C12—S1—O4	−143.3 (3)
C2—C1—C8—C7	−178.9 (5)	C17—C12—S1—N2	−84.7 (4)
C6—C1—C8—C7	1.1 (5)	C13—C12—S1—N2	100.2 (3)
C2—C1—C8—C9	−1.7 (7)	C1—C6—N1—C7	−1.2 (5)
C6—C1—C8—C9	178.2 (4)	C5—C6—N1—C7	179.6 (5)
C7—C8—C9—C10	103.7 (5)	C8—C7—N1—C6	2.0 (6)
C1—C8—C9—C10	−72.8 (5)	C11—C10—N2—S1	−104.3 (3)
C8—C9—C10—N2	−62.1 (4)	C9—C10—N2—S1	134.4 (3)
C8—C9—C10—C11	175.6 (3)	O3—S1—N2—C10	36.3 (3)
N2—C10—C11—O1	−31.5 (6)	O4—S1—N2—C10	167.5 (3)
C9—C10—C11—O1	90.8 (5)	C12—S1—N2—C10	−76.9 (3)
N2—C10—C11—O2	151.1 (4)	C12—C17—N3—O5	48.9 (6)
C9—C10—C11—O2	−86.6 (4)	C16—C17—N3—O5	−130.7 (5)
C17—C12—C13—C14	1.7 (6)	C12—C17—N3—O6	−132.4 (5)
S1—C12—C13—C14	177.1 (3)	C16—C17—N3—O6	48.0 (6)

### Hydrogen-bond geometry (Å, º)

**Table:**

*D*—H···*A*	*D*—H	H···*A*	*D*···*A*	*D*—H···*A*
N1—H1···*Cg*1^i^	0.86	2.77	3.565 (4)	155
N2—H2···O1^ii^	0.86	2.10	2.918 (4)	158

Symmetry codes: (i) *x*−1/2, −*y*+3/2, −*z*+3/4; (ii) *y*, *x*, −*z*+1.

## References

[bb1] Bruker (2007). *APEX2* and *SAINT* Bruker AXS Inc., Madison, Wisconsin, USA.

[bb2] Deng, X. & Mani, N. S. (2006). *Green Chem.* **8**, 835–838.

[bb3] Farrugia, L. J. (1997). *J. Appl. Cryst.* **30**, 565.

[bb4] Flack, H. D. (1983). *Acta Cryst.* A**39**, 876–881.

[bb5] Khan, I. U., Arshad, M. N., Mubashar-ur-Rehman, H., Harrison, W. T. A. & Ali, M. B. (2011*a*). *Acta Cryst.* E**67**, o2325. 10.1107/S1600536811032089 PMC3200826 22058950

[bb6] Khan, M. H., Khan, I. U., Arshad, M. N., Rafique, H. M. & Harrison, W. T. A. (2011*b*). *Crystals*, **1**, 69–77.

[bb7] Sheldrick, G. M. (2008). *Acta Cryst.* A**64**, 112–122. 10.1107/S0108767307043930 18156677

[bb8] Spek, A. L. (2009). *Acta Cryst.* D**65**, 148–155. 10.1107/S090744490804362X PMC2631630 19171970

